# Ferroptosis is Involved in the Pharmacological Effect of Ginsenoside

**DOI:** 10.2174/0113895575277359231210145922

**Published:** 2024-01-11

**Authors:** Juling Feng, Haodong Chen, Yangbo Liu, Qidi Ai, Yantao Yang, Wenbin He, Lei Zhao, Shifeng Chu, Naihong Chen

**Affiliations:** 1 Hunan University of Chinese Medicine & Hunan Engineering Technology Center of Standardization and Function of Chinese Herbal Decoction Pieces, Changsha, 410208, Hunan, China;; 2 State Key Laboratory of Bioactive Substances and Functions of Natural Medicines, Institute of Materia Medica & Neuroscience Center, Chinese Academy of Medical Sciences and Peking Union Medical College, Beijing, 100050, China;; 3 Research lab of translational medicine, Hengyang Medical College, University of South China, Hengyang, Hunan 421001, China;; 4 Shanxi Key Laboratory of Traditional Chinese Medicine Encephalopathy, Shanxi University of Traditional Chinese Medicine, Taiyuan, 030024, China

**Keywords:** Ginsenoside, ferroptosis, pharmacological target, iron metabolism, lipid metabolism, amino acid metabolism

## Abstract

Ginsenoside is the principal active ingredient in ginseng. Several investigations have found that ginsenosides have anti-inflammatory, antioxidant, anti-apoptotic, anti-cancer, and anti-allergic activities. Ferroptosis is an iron-dependent, non-apoptotic form of cell-regulated death caused by lipid peroxidation. Iron, lipid, and amino acid metabolism orchestrate the complex ferroptosis response through direct or indirect regulation of iron accumulation or lipid peroxidation. More and more research has demonstrated that ginsenoside impacts illnesses *via* ferroptosis, implying that ferroptosis might be employed as a novel target of ginsenoside for disease therapy. This article examines the molecular mechanism of ferroptosis as well as the current advancement of ginsenoside in influencing disorders *via* ferroptosis.

## 1 INTRODUCTION

Ginseng is the dried root of the Araliaceae plant *Panax ginseng C. A. Mey* [1]. For thousands of years, they have mostly been utilized to cure weakness and exhaustion in East Asia and North America [2]. The primary biologically active components of ginseng are ginsenosides, which are obtained from the plant's roots, leaves, stems, fruits, and flower heads. Asian ginseng (*Panax ginseng*), Siberian ginseng (*Eleutherococcus senticosus*), American ginseng (*Panax quinquefolius*), and Indian ginseng (*Withania somnifera*) are the most popular ginseng species utilized in medical materials. Ginsenosides have the structure of triterpenoid saponins. According to studies, ginsenosides have a variety of pharmacological properties that can be used to treat a variety of disorders, including those that are anti-inflammatory, immunomodulatory, antioxidant, and anticancer.

Ferroptosis is an iron-dependent, non-apoptotic kind of cell death that varies from apoptosis, pyroptosis, and necrosis in terms of appearance, genetics, metabolism, and molecular biology [3]. Ferroptosis is characterized by an excess of lipid peroxides (LPs) and iron-dependent reactive oxygen species (ROS) [4-6]. While the iron-chelating medicines deferoxamine and deferiprone can inhibit ferroptosis because they lower intracellular iron content, ferrostatins and liproxstatins can reduce lipid peroxidation to prevent ferroptosis [7]. By producing lipid hydroperoxides, the iron-containing enzyme lipoxygenase actively promotes ferroptosis. Its activity is reliant on the stimulation of long-chain fatty acid CoA ligase 4 (ACSL4)-dependent lipid biosynthesis [8].

Ferroptosis has emerged as a research focus as a relatively recent way of regulating cell death. Ferroptosis has been demonstrated to be important for cellular homeostasis maintenance and to play a role in a number of pathological processes, including tumor growth, neurological disorders, and ischemia damage [7, 9-12]. Ferroptosis may be used as a drug target for the treatment of a variety of diseases. Its mechanism has been discovered to primarily reduce iron accumulation, remove lipid peroxides, and inhibit GPX4. Iron-chelating drugs (*e.g.,* deferoxamine and deferiprone) can decrease intracellular iron accumulation and are used to treat heart disease [13, 14], cancer [15], neurodegenerative illnesses [16-18], and neuronal damage [19]. Small- molecule medications used to treat neurodegenerative illnesses, such as liproxstatin 1 (Lip-1) and vitamin E, reduce ferroptosis chiefly by eliminating LPs [20]. N-acetylcysteine modulates GPX4 and is used to treat Parkinson's disease [21, 22]. Activated ACSL4 contributes to tissue injury during intestinal ischemia/reperfusion through ferroptosis [10]. Ginsenoside can cure diseases by acting on a variety of pharmacological targets. Recent research has shown that ginsenoside can be utilized to treat disorders by employing ferroptosis as a possible therapeutic target. In this study, we examine the basic mechanisms of ferroptosis and the mechanisms by which ginsenoside regulates ferroptosis to provide evidence that ferroptosis is a therapeutic target for ginsenoside.

## 2 SEARCH STRATEGY

From 2001 to 2023, we assessed over 200 articles and cited 92 for their relevance and innovation to the topic, including 19 reviews and 73 research papers. The search keywords were: “ginsenosides AND ferroptosis”, “ginsenosides”, ” ferroptosis “, “ginsenosides AND chemistry “, “ginsenosides AND drug targets”, ” ferroptosis AND drug targets”, ” ferroptosis AND iron metabolism”, ” ferroptosis AND lipid metabolism” and ” ferroptosis AND amino acid metabolism” in all fields. We searched the databases PubMed, Sci-Hub, Science Direct, PMC, and Web of Science to identify relevant papers.

## 3 MANIFESTATION OF FERROPTOSIS

**Fig. (1):**
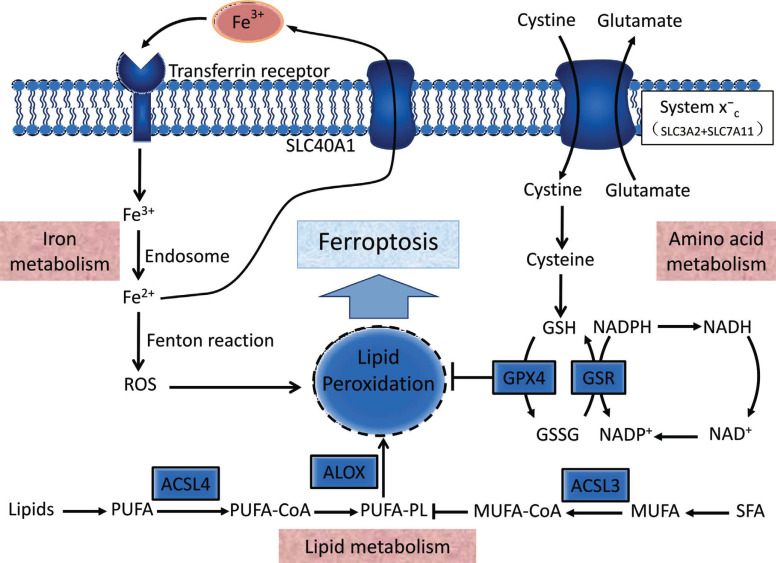
The primary molecular processes of ferroptosis. Iron metabolism, amino acid metabolism, and lipid metabolism all play essential roles in the development of ferroptosis. Excess Fe^3+^ binds to transferrin and forms endosomes *via* the transferrin receptor during iron overload. It is converted to Fe^2+^ by enzymes and then reaches the cytoplasm. The Fenton reaction occurs to facilitate ROS generation, enhance lipid peroxidation, and result in ferroptosis. System XC-mediated cystine metabolism can generate glutathione (GSH) and activate glutathione peroxidase 4 (GPX4) to reduce lipid peroxidation, sparing cells against ferroptosis. The lipid metabolic balance between MUFA-PLs and PUFA-PLs determines whether or not ferroptosis occurs. Under the influence of enzymes, lipids and saturated fatty acids (SFA) produce poly- and mono-saturated fatty acids (PUFA and MUFA). By combining coenzyme A with fatty acids and employing ACSL enzymes (ACSL4 and ACSL3), PUFA-CoA can promote PUFA-PL, whereas MUFA-CoA can inhibit PUFA-PL. Lipoxygenase (*e.g.*, ALXO) catalyzes lipid peroxidation and promotes ferroptosis by acting on PUFA-PL.

Ferroptosis is strongly linked to iron metabolism, lipid metabolism, and amino acid metabolism (Fig. **1**). ROS was assumed to be the principal promoter of ferroptosis for a long time; however, it was subsequently shown that ROS-independent signals may also trigger ferroptosis [23, 24]. However, ROS can undoubtedly cause ferroptosis.

### 3.1 Iron Metabolism

Iron is necessary for cell viability; nevertheless, iron deficiency and excess can result in pathological illnesses. However, iron overload that is brought on by abnormal iron intake, transport, and storage can result in ferroptosis [25].

Transferrin (TF) and transferrin receptor (TFRC) are crucial for iron transport in the body. TF and TFRC dysfunction can result in iron deficiency and iron overload. TF and TFRC knockouts prevent drug-induced ferroptosis [26, 27]. Ferrireductases from the cell surface, including cytochrome B reductase 1 (CYBRD1), and the release of cellular reductants, like ascorbate, transform non-TF-bound iron (NTBI) into the ferrous state. Then, it is transported into cells by transmembrane transporters, such as solute carrier family 11 member 2 (SLC11A2), solute carrier family 39 member 8 (SLC39A8/ZIP8), or solute carrier family 39 member 14 (SLC39A14/ZIP14) [28, 29]. The expression of TFRC and SLC11A2 mRNA increased in HT1080 cells after erasin administration to induce ferroptosis [30]. Fe^2+^ is released within the cell as cytosolic heme oxygenase 1 (HMOX1) processes heme, and hemin is an inducer of HMOX1 production. Hemin can induce ferroptosis, but HMOX1 can prevent ferroptosis in hepatocellular carcinoma and radiation-induced lung injury [31-33]. NFS1 cysteine desulfurase can synthesize heme and iron-sulfur clusters from iron transported to mitochondria and suppress ferroptosis in lung cancer cells produced by oxidative stress damage [34]. Ferritin is vital in preventing iron-mediated oxidative damage. In neuroblastoma cells, mitochondrial ferritin suppresses erastin-induced ferroptosis [35]. Ferritin heavy chain 1 mutation can induce ferroptosis in Drosophila, resulting in growth and development defects in Drosophila [36]. The only transmembrane exporter of non-heme iron is the solute carrier family 40 member 1 (SLC40A1). SLC40A1 overexpression prevented ferroptosis, whereas SLC40A1 knockdown increased ferroptosis [37, 38]. Ceruloplasmin (CP), as a ferroxidase, may control ferroptosis by using the iron export protein SLC40A1. By boosting intracellular ferrous concentration and lipid peroxidation *via* SLC40A1, CP depletion can suppress erastin- and RSL3-induced ferroptosis in HCC cells [39].

### 3.2 Lipid Metabolism

There is no doubt that lipid peroxidation is a hallmark of ferroptosis. Lipid peroxidation requires complex lipid metabolic processes. Physiologically, intracellular phospholipids (PLs) containing polyunsaturated fatty acyl (PUFA) chains are converted into phospholipid peroxides (PLOOH) by both enzymatic and non-enzymatic lipid peroxidation with the help of bioactive iron. However, when PLOOH cannot be effectively neutralized, it can accumulate in the cell, destroying the integrity of the plasma membrane, and the cell undergoes ferroptosis. ACSL4 is an important biological enzyme to produce PUFA-PLs and is also an important regulator of ferroptosis. In fact, lipid synthesis, storage, utilization, and peroxidation all play important roles in the regulation of ferroptosis.

Sterol regulatory element binding protein-1 (SREBP-1) regulates the expression of many lipid-synthesis enzymes, including acetyl-CoA Carboxylase (ACAC), fatty acid synthase (FASN), and SCD1. Knockdown of SREBP1 or SCD1 induced ferroptosis in cancer cells [40, 41], and the ACAC inhibitor 5-(tetradecyloxy)-2-furoic acid (TOFA) prevent ferroptosis [42]. Desaturases (FADS1/2) and elongation enzymes (*e.g.*, ELOVL5) are involved in the synthesis of long-chain PUFAs; hence, FADS1, FADS2, and ELOVL5 all contribute to ferroptosis [43]. ACSF2 (acyl-CoA synthetase family member 2) and CS (citrate synthesis enzymes) are involved in fatty acid metabolism in mitochondria, and knockdown of ACSF2 or CS inhibited erastin-induced ferroptosis [3]. The formation and disintegration of lipid droplets modulate the concentration of intracellular fatty acids (PUFA *vs*. MUFA), altering cell vulnerability to ferroptosis. CD36 facilitates fatty acid entrance into the cell and increases PUFA content, resulting in ferrocytosis of B1 and marginal zone cells and CD8+T cell infiltration into tumor tissue [44-46]. Etomoxir, an inhibitor of carnitine palmitoyltransferase 1 (CPT1), a critical enzyme in the degradation of fatty acids, increased RSL3-induced ferroptosis [47]. 2,4-dienoyl-CoA-reductase 1 (DECR1) is a rate-limiting enzyme in polyunsaturated fatty acid reduction. DECR1 knockdown increases intracellular PUFA concentration and promotes ferroptosis in cancer cells [48, 49].

As the core event in the occurrence of ferroptosis, the initiation, propagation, and termination of lipid peroxidation all affect ferroptosis.

Lipid peroxidation can occur both enzymatically and non-enzymatically. The Fenton reaction of labie iron with hydrogen peroxide (H_2_O_2_) and PLOOH produces hydroxyl radicals, peroxyl radicals, lipid hydroxyl radicals PLO˙, and peroxyl radicals PLOO˙. As a result, intracellular iron levels can influence lipid peroxidation and govern ferroptosis. Cells are more susceptible to ferroptosis when the iron exporter ferroportin is inhibited [50, 51]. ​Lipids can be peroxidized by various oxygenases to generate lipid-free radicals. ALOX is a non-heme ferric oxide enzyme that can directly or interact with PEBP1/RKIP (phosphatidylethanolamine binding protein 1) to oxidize PUFA-PE to initiate ferroptosis [47, 52]. ALOX suppression inhibited ferroptosis, but ALOX knockdown did not inhibit RSL3-induced ferroptosis [53]. However, the expression of the ALOX gene was not detected in the cell lines studied for ferroptosis [54]. Thus, the ALOX enzyme cannot act independently on ferroptosis. Cytochrome p450 oxidoreductase (POR) is a lipid peroxidation oxygenase. POR may facilitate ferroptosis [55]. GPX inhibited lipid peroxidation by converting PLOOH to a non-toxic PL alcohol (PLOH). The conditional knockdown of GPX4 caused ferroptosis [56].

### 3.3 Amino Acid Metabolism

Both the synthesis of glutamine and the metabolism of amino acids have been connected to ferroptosis [57].

The cysteine-glutamate antiporter transport system (system Xc-)/cysteine (Cys)/ GSH/ GPX4 may impact ferroptosis by scavenging LPs. System Xc- may convert extracellular cystine to cysteine, whereas ferroptosis inducer erastin can inhibit system Xc-, reducing intracellular cysteine. Low cystine levels can deplete glutathione, inactivating GPX4, producing ROS, and triggering ferroptosis [3, 58, 59]. CBS (cystathionine beta-synthase) and CTH (cystathionine gamma-lyase) may generate cysteine *via* the transsulfuration pathway, but CBS or CTH inhibitors can trigger ferroptosis in hepatoma cells or NSC-34 motor neuron-like cells [60, 61]. Cysteine dioxygenase (CDO1) catabolizes Cys to taurine, and CDO1 deletion increases intracellular GSH levels, inhibits ROS and LP, and prevents ferroptosis [62, 63]. BCAA can decrease ROS generation by generating GSH, but BCAA 2 can limit ferroptosis by antagonizing systemXc- and inhibiting ferroptosis in liver cancer and pancreatic cancer cells [64]. A tryptophan metabolite called indole-3-pyruvate scavenges free radicals and promotes antioxidant processes, trapping lipid peroxyls and inhibiting ferroptosis [65].

Glutamine metabolism is involved in the regulation of ferroptosis. Glutaminase enzymes (GLS1 and GLS2) may convert glutamine to glutamate. Glutamine may be a ferroptosis inducer [25], while GLS2 suppresses ferroptosis by decreasing intracellular LP [66].

### 3.4 Other Metabolic Pathways Associated with Ferroptosis

Coenzyme Q10 can prevent ferroptosis due to its antioxidant action in the cell membrane [67]. HMG CoA is the rate-limiting enzyme in the mevalonate route of lipid metabolism, and certain statins can impede its activity, lowering coenzyme Q10 and inhibiting GPX4 production, causing ferroptosis [67, 68]. NADPH is a powerful intracellular oxidizing reductant that may remove LPs during ferroptosis. NADPH can also be employed as a ferroptosis biomarker in tumor cell lines [69]. NADPH levels are reduced by ferroptosis inducers Erastin, RSL3, and FIN56 [69]. The susceptibility to ferroptosis was raised when NADPH was knocked down [70]. Selenium is a trace element that is necessary for the human body and has antioxidant properties. Because selenium is also necessary for the manufacture of GPX4, selenium supplementation prevents ferroptosis, but selenium deficiency promotes it [71].

## 4 THE RELATIONSHIP BETWEEN GINSENOSIDES AND FERROPTOSIS

**Fig. (2):**
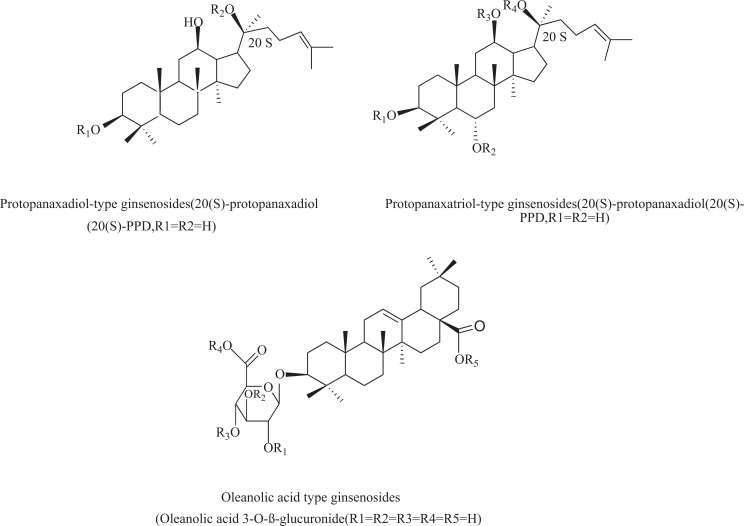
The structure of ginsenosides.

Ginsenosides are divided into three categories based on their aglycone: protopanaxadiol-type saponins (Ra1, Ra2, Ra3, Rb1, Rb2, Rb3, Rc, Rd, Rg3, Rh2, *etc*.), protopanaxatriol-type saponins (Re, Rg1, Rg2, Rf, Rh1, *etc*.), and oleanane-type saponins (Ro) (Fig. **2**). Dammarane-type saponins are tetracyclic triterpene saponins that include protopanaxadiol and protopanaxatriol, whereas oleanane-type saponins are pentacyclic triterpene saponins [72]. More than 90% of the total saponins found in ginseng roots come from the ginsenosides Rb1, Rb2, Rc, Rd, protopanaxtriol Re, Rg1, and the malonyl-ginsenoside derivatives [73]. Additionally, ginseng can change during processing into other ginsenosides, such as ginsenosides Rg5 and Rg6, which are dehydrated forms of Rg3 and Rg2, respectively [72]. In actuality, it has been shown that the benefits of ginseng vary depending on the processing technique. The many ginseng metabolites and their biological effects have been further described using contemporary tools and techniques. Typically, ginseng is consumed orally. Under the influence of gastrointestinal gastric acid, intestinal microorganisms, and various digestive enzymes, ginsenosides can change into ginsenosides with numerous structural variations. These metabolized ginsenosides frequently exhibit more potent biological effects [72]. A typical chemical, compound K, was created *via* the metabolism of the protopanaxadiols Rb1, Rb2, and Rc through the human intestinal flora using bacterial glucosidase enzymes [74, 75]. It is worth noting that various ginsenosides can follow distinct metabolic routes as they move through the stomach and intestines after oral ingestion. When exposed to stomach acid, Rb1, Rb2, and Rc degrade Rg3. When Rg3 reaches the human digestive tract, it can break down into Rh2 [76]. Additionally, Rg1 hydrolyzes in the stomach to produce Rh1, and once Rg1 reaches the colon, intestinal flora converts Rg1 into ginsenoside F1 [74].

Most diseases, particularly chronic diseases such as hypertension and diabetes, are still major medical problems around the world, and their treatment remains a major focus of effort. Although a huge number of new modern pharmaceuticals are being developed at a rapid pace, they frequently have a particular target and serious adverse effects. The original and pure active ingredients of natural products frequently have multi-targeting, high efficiency, and low side effects and can reduce the risk of chronic diseases [77, 78]. Ginsenosides, a powerful component of the natural substance ginseng, have significant therapeutic potential. There is emerging evidence that they can influence how cells and illnesses evolve through modulating ferroptosis (Table **1**). Rd is a protopanaxadiol saponin, and the results indicated that CCl4 treatment considerably decreased GSH and GPX4 expression in the liver [79], but ginsenoside Rd treatment significantly boosted their expression. Ginsenoside Rd inhibits the cGAS/STING signaling pathway, therefore protecting mice against ccl4-induced acute liver injury (ALI) [80]. IKE, a ferroptosis inducer, has the potential to undo the beneficial effects of ginsenoside Rd on liver pathology [80]. Rg3 is a protopanaxadiol-type saponin that can lower intracellular lipid ROS, MDA, and Fe^2+^ levels, enhance GSH content, and protect cells against ferroptosis in pancreatitis [81]. As a consequence, a combined ferroptosis and apoptosis therapy using a ginsenoside Rg3-modified nanocatalyst to initiate both processes has been developed to prevent pancreatic cancer development [82]. Ginsenoside Re, which belongs to the 20(S)-propanaxtriol group, has the ability to up-regulate the major component of the cystine/glutamate antiporter SLC7A11, enhance glutathione production, and minimize ferroptosis caused by cardiac ischemia-reperfusion [83]. Ginsenoside Re increases GPX4 expression by activating PI3K/Akt and ERK, decreases phospholipid peroxidation, and protects neurons from oxidative stress damage [84]. In rats with sepsis-induced acute kidney injury (SI-AKI) and human renal tubular epithelial cells (HK-2) with LPS-induced ferroptosis, ginsenoside Rg1 can reduce lipid peroxidation and ferroptosis by reducing iron content, FTL, FTH, and MDA levels while increasing GPX4, FSP1, and GSH levels. Furthermore, FSP1 knockdown abolished the inhibitory effect of ginsenoside Rg1 on ferroptosis. Ginsenoside Rg1 can thereby lessen SI-AKI by decreasing HK-2 ferroptosis *via* FSP1 [85]. In an allergic asthma mouse model, ginsenoside prodiol metabolite compound K (CK) can lower Th2 cytokines and IgE production, block mast cell activation and goblet cell proliferation, reduce airway hyperresponsiveness and inflammatory response, reduce ROS and MDA content, and upregulate SLC7A11 and GPX4. As a result, CK can suppress ferroptosis and act as an anti-asthmatic. RH3, a metabolite of the ginoside Rg5, can decrease the production of SLC7A11 and GPX4 by disrupting the Stat3/p53/NRF2 signaling pathway, resulting in GSH depletion and the buildup of iron, lipid ROS, and MDA, ultimately leading to ferroptosis of colorectal cancer cells [86]. Secondary ginsenoside Rh4 has been shown to raise the content of ROS in colorectal cancer cells, up-regulate the production of autophagy-related proteins, activate autophagy, increase the amounts of lipid ROS, iron, and MDA, and decrease the content of GSH, resulting in ferroptosis [87].

In conclusion, ginsenosides influence disease ferroptosis *via* iron and amino acid metabolism. As a result, therapeutic ginsenoside targets critical iron and amino acid metabolism components, including ROS and GPX4, and can be employed to better treat ferroptosis. Combination therapy may be more effective while requiring less effort since different ginsenosides target distinct targets. Each disease has multiple death modes, and a new study has found that ginsenosides can help regulate these various cell death modes. Ginsenoside, a naturally occurring ferroptosis treatment, is extremely effective, has a broad spectrum of activity, and has few side effects. Most ginsenosides, including Rg1, Rb1, Rb2, and Rd, have antioxidant properties among their many biological functions [88-92], and antioxidant activity is important in ferroptosis. More evidence that various ginsenosides can be used as therapeutic targets to treat ferroptosis-related illnesses is expected to emerge as research progresses.

## CONCLUSION

Ferroptosis is primarily caused by two mechanisms: iron overload and the inactivation of GPX4. Ferroptosis is an iron-dependent form of cell death, and iron excess has been linked to a number of disorders. During an acute myocardial infarction, an excess of iron can cause myocardial cell death. Regulating intracellular iron levels to limit myocardial cell ferroptosis might thereby lessen myocardial cell damage and improve heart disease prognosis. Ginsenosides have been discovered to alter illness by controlling intracellular iron concentration and ferroptosis. As a result, ginsenoside can be utilized to treat disorders by regulating intracellular iron. As key intracellular antioxidants, GPX4 and GSH protect cells from ferroptosis. Thus, GSH deficiency and GPX4 inactivation result in ferroptosis. The antioxidant system System Xc/GSH/GPX4 can be employed as a therapeutic target for illness therapy. In fact, blocking the main components in the System Xc/GSH/GPX4 antioxidant system has been shown to promote ferroptosis in tumor cells, which can be utilized to treat cancers, particularly drug-resistant tumors. Ginsenoside, in fact, inhibited GPX4, causing ferroptosis in colorectal cancer cells. Hence, ginsenoside exhibits potential as a therapeutic agent targeting the key constituents of system Xc/GSH/GPX4 for disease management. However, ferroptosis is a complex process with multiple pathways and molecular targets, and the molecular targets that play a role in different diseases have their own focuses, so inhibiting a single pathway has significant limitations. Ginsenoside has numerous targets and can work on various pathways at the same time to control ferroptosis, which can overcome the limits of single-medication treatment. As a result, multi-target control of ferroptosis by ginsenosides for disease therapy is a significant path for future therapeutic development.

A systematic review of the relationship between ginsenosides, ferroptosis, and diseases can lay the groundwork for further research into the relationship between ginsenosides and ferroptosis in diseases, opening up new avenues for the development of new high-efficiency, low-toxicity drugs with novel mechanisms or drug targets.

## Figures and Tables

**Table 1:** The relationship between ginsenosides and ferroptosis.

**Ginsenosides**		**Target**	**Function**	**Disease**
Protopanaxadiol-type saponins	Rd	GSH, GPX4	Amino acid metabolism	Liver injury
	Rg3	Lipid ROS, MDA, Fe^2+^^, GSH^	Iron and am-ino acid metabolism	Pancreatic disease
Protopanaxatriol-type saponins	Re	SLC7A11, GPX4	Amino acid metabolism	Cardiac ischemia-reperfusion injury, neuron injury
	Rg1	Iron content, FTL, FTH, GPX4, GSH	Iron and am-ino acid met-abolism	Sepsis-induced acute kidney injury
Secondary sapo-nins	Compo-und K	SLC7A11, GPX4, ROS	Iron and am-ino acid met-abolism	Allergic asthma
	RH3	SLC7A11, GPX4,GSH, ROS	Iron and am-ino acid met-abolism	Colorectalcancer cells
	Rh4	ROS, iron, GSH	Iron and am-ino acid met-abolism	Colorectal cancer

## LIST OF ABBREVIATIONS

LPs: Lipid Peroxides
ROS: Reactive Oxygen Species
GSH: Generate Glutathione
GPX4: Glutathione Peroxidase 4
SFA: Saturated Fatty Acids
